# Impacts of Intensive Logging on the Trophic Organisation of Ant Communities in a Biodiversity Hotspot

**DOI:** 10.1371/journal.pone.0060756

**Published:** 2013-04-10

**Authors:** Paul Woodcock, David P. Edwards, Rob J. Newton, Chey Vun Khen, Simon H. Bottrell, Keith C. Hamer

**Affiliations:** 1 School of Biology, University of Leeds, Leeds, United Kingdom; 2 School of Tropical and Marine Biology, James Cook University, Cairns, Australia; 3 School of Earth and Environment, University of Leeds, Leeds, United Kingdom; 4 Sepilok Forest Research Centre, Sabah Forestry Department, Sandakan, Malaysia; University of Guelph, Canada

## Abstract

Trophic organisation defines the flow of energy through ecosystems and is a key component of community structure. Widespread and intensifying anthropogenic disturbance threatens to disrupt trophic organisation by altering species composition and relative abundances and by driving shifts in the trophic ecology of species that persist in disturbed ecosystems. We examined how intensive disturbance caused by selective logging affects trophic organisation in the biodiversity hotspot of Sabah, Borneo. Using stable nitrogen isotopes, we quantified the positions in the food web of 159 leaf-litter ant species in unlogged and logged rainforest and tested four predictions: (i) there is a negative relationship between the trophic position of a species in unlogged forest and its change in abundance following logging, (ii) the trophic positions of species are altered by logging, (iii) disturbance alters the frequency distribution of trophic positions within the ant assemblage, and (iv) disturbance reduces food chain length. We found that ant abundance was 30% lower in logged forest than in unlogged forest but changes in abundance of individual species were not related to trophic position, providing no support for prediction (i). However, trophic positions of individual species were significantly higher in logged forest, supporting prediction (ii). Consequently, the frequency distribution of trophic positions differed significantly between unlogged and logged forest, supporting prediction (iii), and food chains were 0.2 trophic levels *longer* in logged forest, the opposite of prediction (iv). Our results demonstrate that disturbance can alter trophic organisation even without trophically-biased changes in community composition. Nonetheless, the absence of any reduction in food chain length in logged forest suggests that species-rich arthropod food webs do not experience trophic downgrading or a related collapse in trophic organisation despite the disturbance caused by logging. These food webs appear able to bend without breaking in the face of some forms of anthropogenic disturbance.

## Introduction

Trophic organisation defines the flow of energy through ecosystems [1], [2] and can have far-reaching effects on ecosystem properties and processes, and on the conservation of biodiversity [3], [4], [5], [6]. Describing and understanding how anthropogenic disturbance affects trophic organisation is therefore a major concern [7], [8]. The impacts of disturbance depend upon any differences in the relative abundance of each species between undisturbed and disturbed ecosystems, combined with any shifts in the position of each species within the food web. However, evidence for such changes is ambiguous and incomplete. For example, large-bodied vertebrates with high trophic positions (species at the top of the food chain) tend to have small population sizes and require large areas for foraging, and are also influenced by variation in prey populations, making them particularly susceptible to anthropogenic pressure [9], [10], [11], [12]. This prediction is supported by analyses on the threat status of mammals [13], but smaller-bodied organisms can also attain high trophic positions (e.g. compare [14] with [15]), and a negative relationship between trophic position and susceptibility to disturbance may not occur when these other taxa and different forms of disturbance are considered [16]. Studies comparing the trophic positions of species in disturbed and undisturbed ecosystems have generated similarly ambiguous results, with decreases [17], increases [14], [18] and no difference in trophic position found between habitats [19], [20].

By collecting data on both the relative abundances and the trophic positions of species, several recent studies have tested community-level hypotheses concerning the impacts of disturbance. The dynamic constraints hypothesis, which predicts that disturbance should reduce food chain length [21], [22], has received particular attention because variation in food chain length influences key ecological processes [23], [24]. Again, however, evidence for a link between disturbance and food chain length is conflicting (see [25], [26] for reviews). Moreover, this evidence comes largely from freshwater ecosystems: in a recent meta-analysis, only one study quantified the effects of disturbance on food chain length in a terrestrial habitat, emphasising the need to broaden the scope of research into the causes and extent of variation in food chain length [26].

Here, we focus on selective logging, which represents the most widespread form of forest disturbance in the tropics, with over 400 million hectares of forest in the permanent timber estate and at least 20% of the tropical forest biome selectively logged between 2000 and 2005 [27]. Selective logging involves the removal of commercially valuable trees above a threshold size, and the process of felling and extracting trees can cause severe residual damage via the labyrinth of logging roads [28], soil compaction, and high mortality of non-harvested trees [29]. These changes lead to significant shifts in abundance across a range of taxa [30], [31], [32], [33] and have prompted urgent calls for a greater understanding of the impacts of logging on the structure and functioning of rainforest ecosystems [34], [35]. We investigate how selective logging affects the relative abundance of species within a tropical rainforest, and we use the numerical measures of trophic positions provided by stable isotope ratios (expressed as δ^15^N; [36], [37]) to quantify any shifts in the trophic positions of species. We then combine these pieces of species-level information to evaluate any shifts in trophic organisation that are associated with logging disturbance.

Our study region is the Sundaland biodiversity hotspot in Southeast Asia [38], [39], which has experienced some of the highest timber extraction intensities globally [40], [41]. We focused on the leaf-litter ant assemblage, because it is highly abundant and diverse, and because ants exert a key influence on ecosystem functioning through several types of trophic interaction that are reflected in the trophic positions of each species (e.g. as seed dispersers and predators [42], [43], [44]). Furthermore, the assemblage has undergone significant shifts in composition following logging, and although many species currently persist [45], the consequences of these changes in composition for trophic organisation have not been studied. Accordingly, we first investigated species-level changes by testing two predictions: (i) there is a negative relationship between the trophic position of a species in unlogged forest and its change in abundance following logging, and (ii) changes in community composition and habitat structure that follow logging are accompanied by shifts in the trophic positions of species that persist in logged forest. We then examined community-level changes by testing two further predictions: (iii) the frequency distribution of trophic positions differs between unlogged and logged forests, and (iv) disturbance reduces food chain length.

## Materials and Methods

### Ethics Statement

All necessary permits were obtained for the described field studies. Approval and permits were provided by Yayasan Sabah, the Danum Valley Management Committee, Sabah Chief Minister’s Department, the Economic Planning Unit of the Prime Minister’s Department and the Sabah Forestry Department.

### Study Site

Fieldwork was conducted within the 1 million ha Yayasan Sabah logging concession in Sabah, Borneo (4° 58′N, 117° 48′E) which is one of the most biologically important areas of lowland rainforest in Borneo [46]. We compared the unlogged forests of the Danum Valley Conservation Area and Palum Tambun Watershed Reserve (45,200 ha) with the contiguous Ulu Segama-Malua Forest Reserve, which is a 238,000 ha area of production forest. Unlogged forests in the concession are dominated by commercially valuable trees of the family *Dipterocarpaceae* [47]. Production forests in the concession have undergone two rounds of selective logging (first rotation: 1987–1991, second rotation: 2001–2007), producing total timber yields of ∼145 m^3^ ha^−1^, some of the highest rates of timber removal globally (see [41] for further details).

### Sampling

In unlogged forest, we established eight transects ≥500 m apart (Figure S1). In logged forest, we grouped transects into four sites, each comprising two transects separated by 500–800 m and spaced such that the unlogged forest was central between two logged sites to the south-east and two logged sites to the north-west. Distances between logged forest transects (28.3±3.7 km) were similar to those between logged and unlogged forest transects (23.6±0.5 km). Ants were sampled from seven 1 m^2^ census points separated by 25 m and on alternate sides of each transect (56 sampling points in each type of forest) using the Winkler method ([48], see [45] for details). The Winkler method is unreliable when the leaf litter and soil are damp, so we did not sample for two days following any heavy rainfall. Minor workers were stored in 95% ethanol, identified to genus using online keys [49], and pre-sorted to morphospecies based on external characteristics. Where possible, morphospecies were assigned species names using published keys, online image resources (www.antbase.net, www.antweb.org), and reference collections at the Natural History Museum (London) and Universiti Malaysia Sabah (Kota Kinabalu). Voucher specimens of each species and morphospecies are housed at the Forest Research Centre, Sabah.

To establish baseline δ^15^N values for each transect [37], pairs of leaves were collected from two understorey plants every 15 m along the transect ( = 20 plants per transect), dried in a plant press, and stored in a sealed dry room [50]. All fieldwork took place between May and September 2007 and May and September 2008: three transects in unlogged forest were sampled for ants and baseline material in 2007 and the remaining 13 transects were sampled for ants and baselines in 2008. There is little seasonal variation in climate within the study region [51], and sampling years were similar in terms of environmental conditions (no mast-fruiting, droughts or floods). Repeat sampling from the same locations also indicates that annual variation in ant community composition is low [52].

### Stable Isotope Analysis

A single isotope analysis was conducted to represent all conspecific worker ants from the same sampling point, because these were considered to be from the same colony and therefore non-independent, and because variation in nitrogen isotope ratio values amongst conspecifics from the same point was low [14], [50], [52]. Ants were prepared by removing gasters [14] and oven-drying at 50°C to a constant mass, and plant material was dried in a plant press and ground into a fine powder using a mixer mill. Samples were analysed on a Eurovector 3028HT elemental analyser coupled to a GV Isoprime continuous flow mass spectrometer. Samples and standards were combusted in pure oxygen (N5.0, BOC, UK) injected into a stream of helium at 1020°C. Water and carbon dioxide were removed from the gas stream using magnesium perchlorate and Carbosorb respectively (Elemental Microanalysis, UK). δ^15^N was calculated as ([R_sample_/R_standard_]−1)×10^3^, where R_sample_ is the ^15^N:^14^N ratio of the sample and R_standard_ is the ^15^N:^14^N ratio of the N_2_ reference gas. Sample δ^15^N values were calibrated against the international δ^15^N_air_ scale using the ammonium sulphate standards USGS-25 (−30.4‰) and USGS-26 (+53.7‰) interspersed every 8–12 samples. In addition, an internal yeast check standard was repeated several times in each column and produced standard deviations of 0.1–0.3‰, with a long-term average of −0.55±0.28‰ (1SD) from 265 analyses across all columns. Ant δ^15^N_air_ values were corrected for baseline variation [37] by subtracting the mean δ^15^N of leaf samples collected from the same transect to give a baseline-corrected value (δ^15^N_BC_): δ^15^N_BC_ = δ^15^N_sample_ – δ^15^N_plant mean for transect_ (see [50] for further details).

### Calculation of Stable Isotope Metrics

Trophic position was calculated as λ+(δ^15^N_BC/_E), where λ is the trophic position of the organism used as the baseline (λ = 1 for plants) and E is the enrichment in δ^15^N per trophic level (for ants, E = 3.0; [53]). Leaf-litter ants span almost the full range of trophic positions within the soil food web, from granivores to specialised predators [54], and so we used the colonies with the highest trophic positions in each forest type (top 5% of colonies) as a measure of the realised food chain length [23], [55]. Although specialised predatory ants might occasionally be consumed, the definition of realised food chain length integrates all energy flow pathways through the food web [23], [25] so such infrequent events have little effect on estimates of food chain length.

### Statistical Analysis

We first tested for a relationship between the trophic position of a species in unlogged forest and its change in abundance by using Linear Mixed Effects models (LME) with ‘ant subfamily’ as a random effect to account for phylogeny [56]. We then investigated whether or not the trophic positions of ants differed between unlogged and logged forest by using a LME (including all isotope analyses) with ‘subfamily’ as a random effect, and ‘species’ nested within subfamily to account for repeated measures. We also used General Linear Models to test for differences in trophic position between unlogged and logged forest in each of the commonest species (n≥10 in both types of forest).

Differences between unlogged and logged forest in the trophic organisation of the assemblage were investigated by using Kolmogorov-Smirnov tests to compare the frequency distribution of trophic positions of colonies in each type of forest. We first examined the location and shape of the distributions, and then focused only on the shape by centring each distribution around a mean of zero. Changes in the location of the distribution would indicate a systematic increase or decrease in trophic positions of the assemblage, whilst changes in the shape of the distribution would indicate that colonies become more or less concentrated within the food web. This approach compares differences in trophic structure without considering species identity. It is thus focused on summarising how each colony contributes to the overall trophic structure of the assemblage. Lastly, we used a LME to compare food chain lengths – defined from the 5% of colonies with the highest trophic positions in each type of forest – between unlogged and logged forest. As with previous analyses, each sample was used as a separate measure of trophic position, and ‘subfamily’ and ‘species’ were treated as nested random effects.

## Results

**Figure 1:**
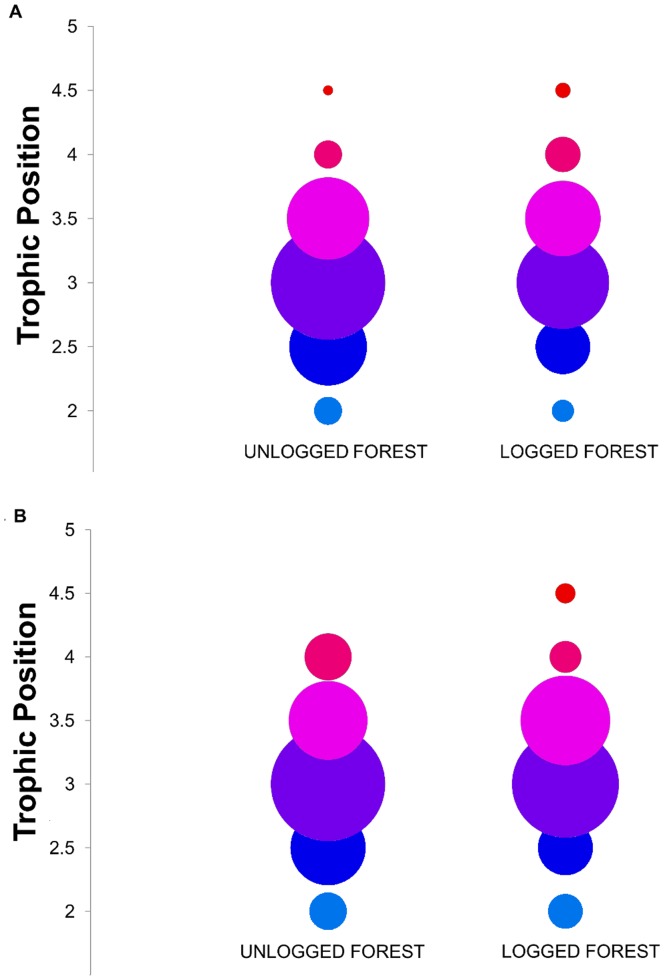
Trophic organisation of ant communities in unlogged and logged forest. Trophic positions of (a) colonies and (b) species in unlogged and logged forest are grouped into trophic categories of 0.5 trophic levels (<2.25, 2.25–2.75, 3.25–3.75 etc.). Bubble sizes represent the proportion of colonies or species in each trophic category for each type of forest (i.e. expressed as a percentage of the total number of colonies [a] or species richness [b] for each forest type).

**Figure 2:**
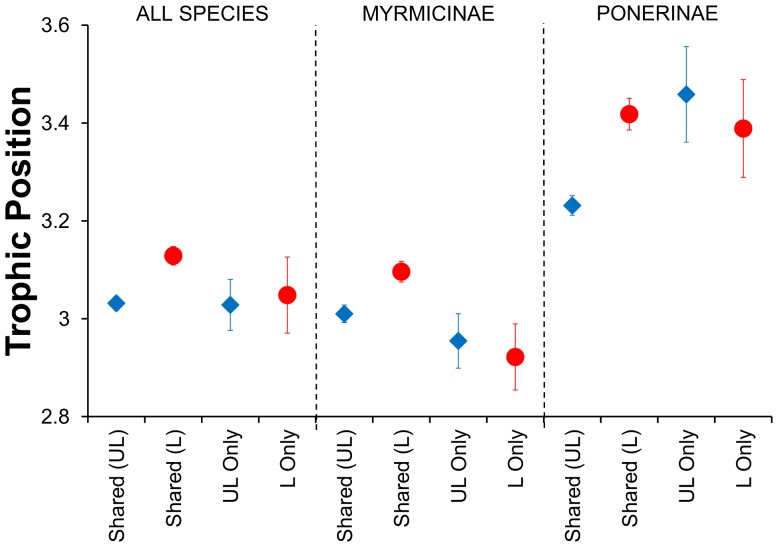
Causes of differences in trophic organisation of ant communities between unlogged and logged forest. Mean trophic positions±S.E. are shown for species found in both unlogged (UL) and logged (L) forest, for species found only in logged forest, and for species found only in logged forest. Means are based on all samples and presented for all species, and for the two commonest ant subfamilies (Myrmicinae and Ponerinae).

We obtained trophic positions for 1427 samples of 159 ant species, comprising 841 samples of 142 species in unlogged forest and 586 samples of 125 species in logged forest. The assemblage was largely carnivorous (mean trophic position of all species = 3.07±0.01 S.E.) but trophic positions of individual species ranged from 2.00–4.43 (Figure 1; Figure S2; Table S1). Trophic positions for ant species sampled in 2007 in unlogged forest did not differ from those for species sampled in 2008 in unlogged forest (2007_mean_ = 3.02±0.02, 2008_mean_ = 3.04±0.02; LME: F_6, 831_ = 0.03, p>0.5). We found no relationship between the trophic position of a species in unlogged forest and its change in relative or absolute abundance (LME: F_1, 44_ = 0.1, p>0.5 in both cases). Furthermore, the mean trophic position of species found only in unlogged forest did not differ from the shared species mean for unlogged forest (LME: F_1, 132_ = 0.05, p>0.5; Figure 2). However, the mean trophic position of species found only in logged forest was marginally significantly lower than the shared species mean for logged forest (LME: F_1, 116_ = 2.76, p = 0.099), suggesting a slight influx of species with trophic positions below the average for logged forest.

**Figure 3:**
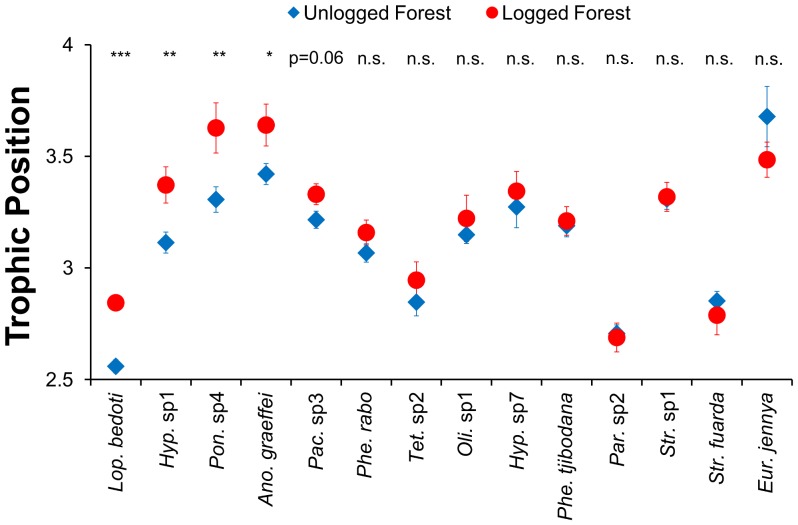
Trophic position of species commonly sampled in unlogged and logged forest. Mean trophic position±S.E. are shown for all species sampled at least 10 times in both types of forest. p values are: *≤0.05, **≤0.01, ***≤0.001. From left to right, species are: *Lophomyrmex bedoti, Hypoponera* sp1, *Ponera* sp4, *Anochetus graeffei, Pachycondyla* sp3, *Pheidole rabo, Tetramorium* sp2, *Oligomyrmex* sp1, *Hypoponera* sp7, *Pheidole tjibodana, Paratrechina* sp2, *Strumigenys* sp1, *Strumigenys fuarda, Eurhopalothrix jennya*.

With respect to prediction (ii), we found strong evidence for increases in trophic positions amongst species that were shared between both types of forest (mean _unlogged forest_ = 3.03±0.01, mean _logged forest_ = 3.13±0.02; LME: F_1, 1197_ = 43.9, p<0.0001; Figure 2). In addition, the trophic positions of four of the 14 most prevalent shared species (n≥10 occurrences in each forest type) were significantly higher in logged forest than in unlogged forest. These four species combined accounted for 12% of all ant occurrences, and we also found a marginally significant increase in a fifth species (Figure 3). As a consequence, the mean trophic position of ants was significantly higher in logged forest than in unlogged forest (LME: F_1, 1261_ = 41.2, p<0.0001) by an average of 0.1±0.03 trophic levels.

The higher trophic positions of species in logged forest translated into shifts in overall trophic organisation, with a significant difference between forest types in the frequency distribution of the trophic positions of colonies (z = 1.86, p = 0.002). When distributions were centred around zero, trophic organisation was marginally significantly different between forest types (z = 1.29, p = 0.071). We also found a significant increase in mean food chain length in logged forest based on the trophic positions of the top 5% of colonies (mean _unlogged forest_ = 3.89±0.04, n = 42 colonies; mean _logged forest_ = 4.10±0.04, n = 29 colonies; LME F _1, 29_ = 10.3, p = 0.0032). This finding was repeatable using the top 10% of colonies (mean _unlogged forest_ = 3.73±0.03, n = 84 colonies; mean _logged forest_ = 3.93±0.03, n = 58 colonies; LME: F_ 1, 91_ = 22.6, p<0.001).

## Discussion

Our results indicate that selective logging significantly alters the trophic organisation of the leaf-litter ant community in tropical rainforests. The change in trophic organisation was not caused by shifts in the relative abundance of different species, but instead was the result of increases in trophic positions amongst species found in both unlogged and logged forest. Higher trophic positions in logged forest resulted in food chains that were 0.2 trophic levels longer and in significant differences between forest types in the distribution of ant colonies through the food web (Figure 1).

### Species-Level Responses to Disturbance

The trophic ecology of most leaf-litter ant species found in tropical rainforests is poorly known, but our results are consistent with knowledge for better-studied species (e.g. *Anochetus graeffei*, *Mystrium camillae*, and *Cerapachys* spp. were highly carnivorous [54]). Also as would be expected, the Ponerinae generally had higher mean trophic positions than the Myrmicinae (Figure 2). We are thus confident that our stable isotope protocol has measured trophic positions accurately, and consequently that the information provided for the many small and cryptic leaf-litter ant species for which diets are largely unknown is also reliable (Table S1).

Increases in trophic positions may result directly from changes in diet or indirectly from changes in the diet of prey species [57]. Both mechanisms probably operate to some degree, although changes in prey diet cannot explain increased trophic positions in ant species that feed exclusively on plant material and/or obligate herbivores. Direct changes in diet are therefore the most likely explanation for the elevation in trophic positions of ant species with a position of <3 in unlogged forest (48 species: Table S1). Although [58] found no difference in the trophic positions of ant genera between forest remnants and pastures, increases in trophic positions in disturbed forest have been detected for weaver ants (*Oecophylla smaragdina*), which switched from the consumption of homopteran exudates and nectar in primary forest to a greater dependency on predation and scavenging in secondary forest regrowth [14]. Furthermore, the higher trophic positions of small mammals in disturbed ecosystems [59] suggest that our findings may be mirrored in other taxa.

Species with high trophic positions are thought to be more susceptible to anthropogenic disturbances such as fragmentation [4], [60] because high trophic position is intrinsically linked to factors such as low population size, high population variability and high dependence on prey populations [9], [10], [12]. However, we found no evidence for a negative relationship between the trophic position of a species in unlogged forest and its change in abundance following logging. This implies that for leaf-litter ants, either the above factors do not influence susceptibility to intensive logging disturbance and/or these factors are not linked to trophic position. Whilst the potential for cascading effects following the loss of large-bodied top predators is a cause for serious concern [6], the prediction that trophic position is an important determinant of susceptibility to anthropogenic impacts thus may not hold generally [16], [61], [62]. This has important conservation implications in terms of understanding what makes a species vulnerable to anthropogenic disturbance, as well as for informing simulations that model the consequences of realistic extinction sequences on food webs (e.g. [63]).

### Community-Level Responses to Disturbance

The differences in trophic positions of individual species translated into significant differences in food chain length between unlogged and logged forests. However, our results are in the opposite direction from that predicted by the dynamic constraints hypothesis [21], [22], with food chains approximately 0.2 trophic levels *longer* in disturbed forest. Nonetheless, our estimates of food chain length for the soil food web in both types of forest (3.73–4.10) are similar to those for other ecosystems (2.70–4.35; [17], [19], [20], [64]). Moreover, the magnitude of change in food chain length falls within the range of values documented by studies used in a recent meta-analysis to test the effects of disturbance [26]. While disturbance can reduce food chain length in some circumstances [17], [65], our study thus supports previous research [19], [20] indicating that disturbance does not have a consistent negative impact on food chain length in complex communities (see also [26]).

Previously documented positive effects of disturbance on food chain length have been attributed to increases in the abundance of early successional organisms [18]. Life history trade-offs mean that these organisms are often more palatable and experience higher herbivory rates [66], [67], and so more energy is available at the base of the food web, which then cascades upwards to lengthen food chains [18]. A similar mechanism could explain our results, with the flush of fast-growing understory plants in regenerating forest [32] providing greater energy to support species at higher trophic positions. In this hypothesis, disturbance is, effectively, influencing food chains by causing changes in local productivity, reflecting the more consistently documented positive relationship between productivity and food chain length [26].

Shifts in trophic organisation also occurred through the rest of the food web, with fewer colonies at low-intermediate trophic levels in logged forest (Figure 1; Figure S2). Whilst inferences of changes in ecosystem functioning following disturbance should be made with caution when direct measurements of the relevant processes are lacking, these results nonetheless imply that the balance between low trophic level functions (e.g. seed dispersal) and high trophic level functions (e.g. predation) in leaf-litter ant communities is modified by anthropogenic disturbance.

### Conclusion

Our results provide strong evidence that trophic structure differs between unlogged forest and forest regenerating from intensive selective logging, and that this difference is caused by changes in the trophic positions of ant species common to both types of forest. Combined with the significant reduction in colony abundance and shifts in community composition detected in heavily logged forest [45], our findings provide further evidence for anthropogenic disruption of the structure and functioning of rainforest ecosystems. Longer-term research will be necessary to determine if and how these changes influence the vulnerability of rainforest food webs to future disturbance (e.g. fire or subsequent logging cycles) and whether or not they are part of a recovery trajectory towards the trophic organisation of undisturbed forests. Importantly, however, there was no evidence for a collapse in trophic organisation similar to that documented following some other forms of anthropogenic disturbance [4]. Our results therefore add empirical evidence to a body of theoretical research [68], [69] suggesting that complex food webs possess a degree of flexibility in the face of some types of anthropogenic disturbance, in this instance effectively bending without breaking.

## Supporting Information

Figure S1 Map of study sites.
(DOCX)

Figure S2 Abundance distributions for ant colonies and ant species in unlogged and logged forest.
(DOCX)

Table S1 Mean trophic positions for ant species in unlogged and logged forest.
(DOCX)
