# Supplementary material for: Impacts of Intensive Logging on the Trophic Organisation of Ant Communities in a Biodiversity Hotspot
Source: PLoS One. 2013 Apr 10;8(4):e60756. doi: 10.1371/journal.pone.0060756 (PMC3622666; doi:10.1371/journal.pone.0060756)

**Figure S1**

**Map of study sites in unlogged and logged forest**. Blue diamonds represent primary forest transects. Red circles represent logged forest sites, each consisting of two transects. Dark grey=unlogged forest, mid-grey=once-logged forest, pale grey=twice-logged (degraded) forest, white=oil palm.


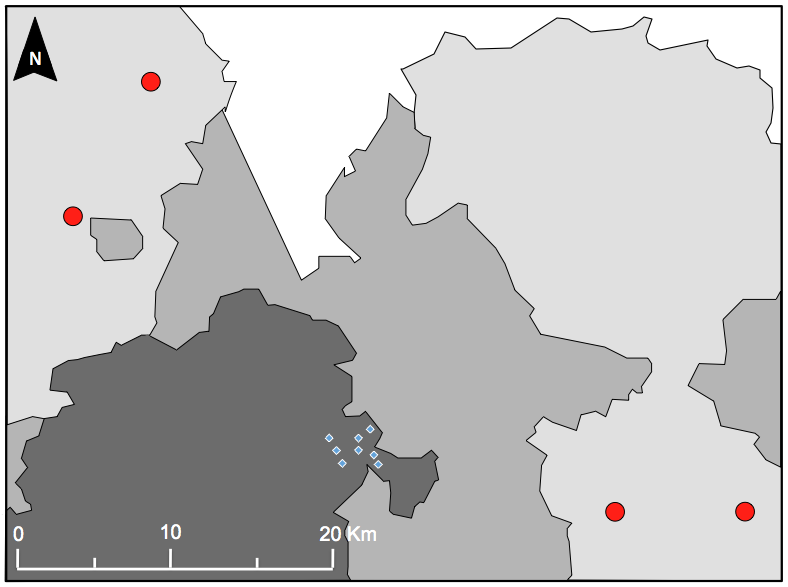

Supplement: Figure S1 — Map of study sites. (DOCX) [file pone.0060756.s001.docx]
