# Supplementary material for: Impacts of Intensive Logging on the Trophic Organisation of Ant Communities in a Biodiversity Hotspot
Source: PLoS One. 2013 Apr 10;8(4):e60756. doi: 10.1371/journal.pone.0060756 (PMC3622666; doi:10.1371/journal.pone.0060756)

**Figure S2**

**Trophic organisation of ant communities in unlogged and logged forest.** Trophic positions of (a) colonies and (b) species in unlogged and logged forest are grouped into trophic categories of 0.5 trophic levels (<2.25, 2.25-2.75, 3.25-3.75 etc.). Bubble sizes represent the number of colonies or species in each trophic category (scaled to reflect the total number of colonies [a] or species [b] for each forest type).


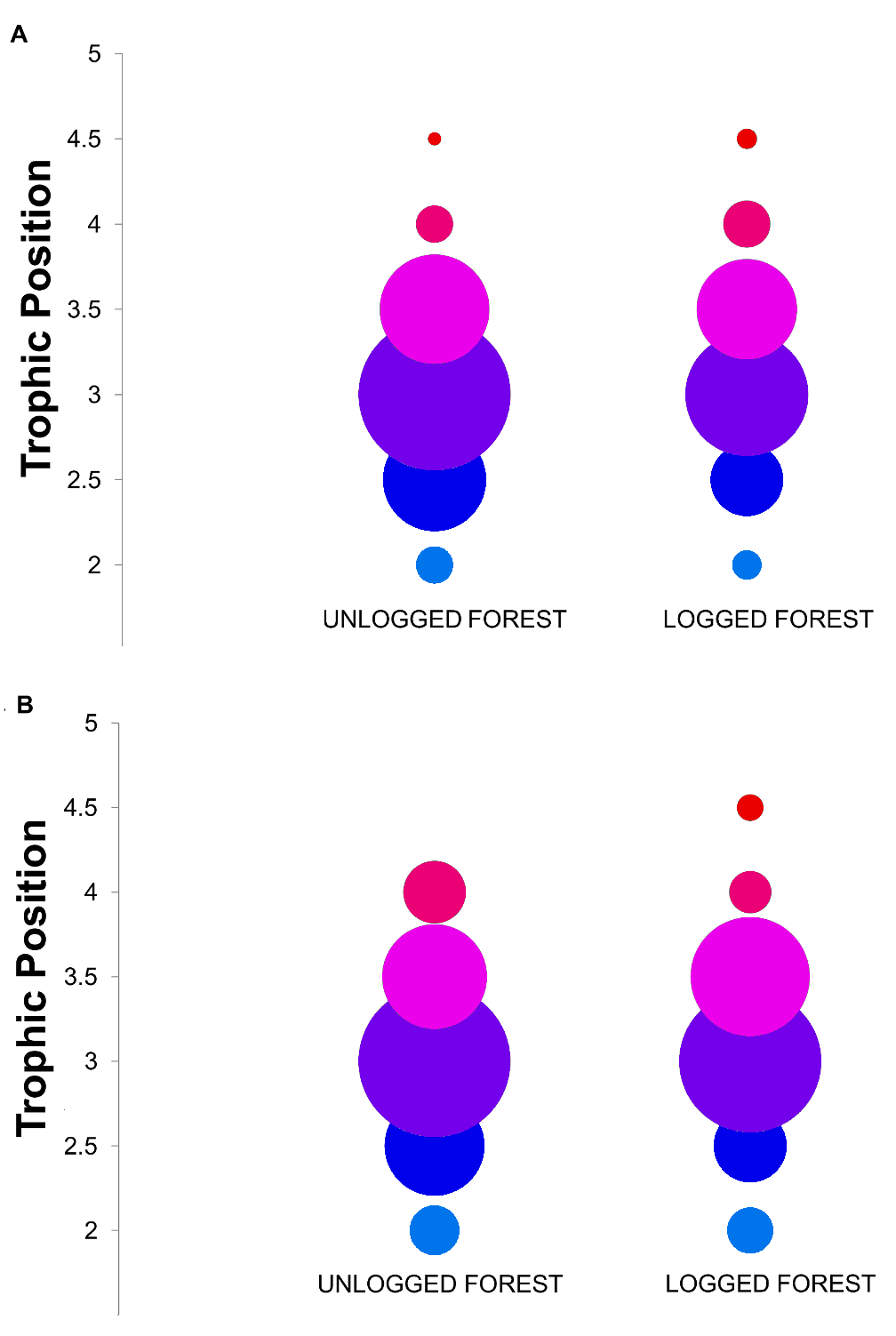

Supplement: Figure S2 — Abundance distributions for ant colonies and ant species in unlogged and logged forest. (DOCX) [file pone.0060756.s002.docx]
