# Supplementary material for: Impacts of Intensive Logging on the Trophic Organisation of Ant Communities in a Biodiversity Hotspot
Source: PLoS One. 2013 Apr 10;8(4):e60756. doi: 10.1371/journal.pone.0060756 (PMC3622666; doi:10.1371/journal.pone.0060756)
Supplement: Table S1 — Mean trophic positions for ant species in unlogged and logged forest. (DOCX) [file pone.0060756.s003.docx]

**Table S1**

Mean trophic position for ant species in unlogged and logged forest.

| **Subfamily** | **Species** | **Unlogged Forest** | **Logged Forest** |
| --- | --- | --- | --- |
| **Ambyloponinae** | *Mystrium camillae* | 3.84 | 4.18 |
|  | *Prionopelta kraepelini* | 3.41 | 3.52 |
| **Cerapachyinae** | *Cerapachys hewitti* |  | 3.72 |
|  | *Cerapachys* sp1 | 3.60 | 3.47 |
|  | *Cerapachys* sp2 | 3.94 |  |
|  | *Cerapachys* sp6 | 3.51 | 3.71 |
|  | *Cerapachys* sp7 | 4.24 | 4.43 |
|  | *Cerapachys* sp10 | 4.05 | 3.84 |
| **Dolichoderinae** | *Dolichoderus thoracicus* | 2.24 | 2.42 |
|  | *Technomyrmex* sp1 | 2.35 | 2.35 |
|  | *Technomyrmex* sp2 | 2.61 | 2.62 |
|  | *Technomyrmex* sp3 | 2.65 |  |
|  | *Technomyrmex* sp4 |  | 2.00 |
| **Ectatomminae** | *Gnamptogenys binghamii* | 3.19 | 2.76 |
|  | *Gnamptogenys costata* |  | 2.91 |
|  | *Gnamptogenys cribrata* | 3.76 | 3.54 |
|  | *Gnamptogenys posteropsis* | 3.21 | 3.20 |
|  | *Gnamptogenys* sp1 | 2.42 | 2.43 |
| **Formicinae** | *Acropyga oceanica* | 2.81 |  |
|  | *Anoplolepis gracilipes* |  | 3.10 |
|  | *Camponotus* sp2 | 2.00 |  |
|  | *Camponotus* sp4 |  | 2.00 |
|  | *Myrmoteras bakeri* | 2.90 | 3.04 |
|  | *Myrmoteras donisthorpei* | 2.71 | 2.85 |
|  | *Myrmoteras iriodum* | 2.98 |  |
|  | *Paratrechina longicornis* | 2.60 | 2.24 |
|  | *Paratrechina* sp1 | 2.40 | 3.66 |
|  | *Paratrechina* sp2 | 2.71 | 2.69 |
|  | *Paratrechina* sp3 | 2.64 | 2.66 |
|  | *Paratrechina* sp4 | 2.47 | 2.76 |
|  | *Paratrechina* sp6 | 2.47 |  |
|  | *Polyrhachis phalerata* | 2.00 | 2.00 |
|  | *Polyrhachis sukarmani* |  | 2.00 |
|  | *Pseudolasius* sp1 | 2.66 | 2.94 |
|  | *Pseudolasius* sp2 | 2.89 | 3.12 |
| **Myrmicinae** | *Acanthomyrmex ferox* | 2.77 | 2.73 |
|  | *Acanthomyrmex* sp1 | 3.04 |  |
|  | *Aphaenogaster* sp | 2.97 | 3.36 |
|  | *Calyptomyrmex near beccarii* |  | 2.91 |
|  | *Calyptomyrmex* sp1 | 3.44 | 3.30 |
| **Subfamily** | **Species** | **Unlogged Forest** | **Logged Forest** |
| **Myrmicinae (contd.)** | *Cardiocondyla* sp | 2.57 |  |
|  | *Crematogaster modiglianii* | 2.51 | 2.72 |
|  | *Crematogaster* sp1 | 2.90 | 2.76 |
|  | *Crematogaster* sp2 | 2.50 | 2.55 |
|  | *Crematogaster* sp5 | 2.23 | 2.22 |
|  | *Crematogaster* sp6 | 2.46 | 2.66 |
|  | *Dacetinops concinnus* | 4.10 | 3.02 |
|  | *Eurhopalothrix dubia* | 3.46 | 3.45 |
|  | *Eurhopalothrix jennya* | 3.68 | 3.48 |
|  | *Lophomyrmex bedoti* | 2.56 | 2.84 |
|  | *Lophomyrmex longicornis* | 2.53 | 2.94 |
|  | *Lordomyrma reticulata* |  | 3.01 |
|  | *Lordomyrma* sp1 | 3.31 | 2.99 |
|  | *Mayriella transfuga* | 2.50 | 3.21 |
|  | *Meranoplus malaysianus* |  | 2.88 |
|  | *Monomorium australicum* | 3.43 | 3.07 |
|  | *Monomorium* sp1 | 2.73 |  |
|  | *Monomorium* sp5 | 2.95 | 2.71 |
|  | *Myrmicaria carinata* | 2.23 |  |
|  | *Myrmecina* sp1 | 2.81 | 3.39 |
|  | *Myrmecina* sp2 | 3.22 | 3.64 |
|  | *Myrmecina* sp3 | 3.20 | 3.47 |
|  | *Oligomyrmex* sp1 | 3.15 | 3.22 |
|  | *Oligomyrmex* sp2 | 3.40 | 3.34 |
|  | *Oligomyrmex* sp3 | 3.24 | 3.01 |
|  | *Oligomyrmex* sp4 | 3.23 |  |
|  | *Pheidole annexus* | 2.93 | 2.90 |
|  | *Pheidole aristotelis* | 2.88 | 3.19 |
|  | *Pheidole cariniceps* | 2.95 | 3.36 |
|  | *Pheidole gombakensis* | 3.39 | 3.47 |
|  | *Pheidole rabo* | 3.07 | 3.16 |
|  | *Pheidole sabahna* |  | 3.01 |
|  | *Pheidole sarawakana* | 3.03 |  |
|  | *Pheidole sauberi* | 2.96 | 3.04 |
|  | *Pheidole spinicornis* | 3.07 | 3.08 |
|  | *Pheidole tjibodana* | 3.19 | 3.21 |
|  | *Pheidole* sp4 | 3.05 | 2.87 |
|  | *Pheidole* sp7 | 3.11 | 3.04 |
|  | *Pheidole* sp8 | 3.12 | 3.34 |
|  | *Pheidole* sp9 | 2.79 | 2.89 |
|  | *Pheidole* sp10 | 3.03 | 3.03 |
|  | *Pheidole* sp12 | 3.27 | 3.09 |
|  | *Pheidole* sp14 | 3.13 | 2.88 |
| **Subfamily** | **Species** | **Unlogged Forest** | **Logged Forest** |
| **Myrmicinae (contd.)** | *Pheidole* sp15 | 2.73 |  |
|  | *Pheidole* sp18 | 3.30 |  |
|  | *Pheidole* sp19 | 2.69 |  |
|  | *Pheidologeton affinis* | 3.12 | 2.87 |
|  | *Pheidologeton pygmaeus* | 2.99 | 2.76 |
|  | *Pristomyrmex rigidus* | 2.99 | 3.12 |
|  | *Proatta butteli* | 3.35 | 3.17 |
|  | *Pyramica mitis* | 2.82 |  |
|  | *Pyramica* sp2 | 3.31 |  |
|  | *Pyramica* sp3 |  | 2.99 |
|  | *Pyramica* sp7 |  | 3.17 |
|  | *Recurvidris browni* | 2.91 |  |
|  | *Recurvidris* sp2 | 2.14 |  |
|  | *Recurvidris* sp4 | 3.08 |  |
|  | *Solenopsis* sp1 | 2.88 | 2.89 |
|  | *Strumigenys amasara* | 3.51 | 3.37 |
|  | *Strumigenys fuarda* | 2.85 | 2.79 |
|  | *Strumigenys ignota* | 3.15 | 3.15 |
|  | *Strumigenys kraepelini* | 4.03 |  |
|  | *Strumigenys lebratyx* | 3.13 | 3.82 |
|  | *Strumigenys near doriae* | 3.23 | 4.30 |
|  | *Strumigenys rotogenys* | 2.72 | 2.53 |
|  | *Strumigenys* sp1 | 3.31 | 3.33 |
|  | *Strumigenys* sp2 | 2.91 | 2.85 |
|  | *Strumigenys* sp7 | 3.06 |  |
|  | *Strumigenys* sp10 | 3.32 | 3.73 |
|  | *Strumigenys* sp12 | 3.18 | 3.74 |
|  | *Strumigenys* sp21 | 2.77 | 2.96 |
|  | *Tetramorium insolens* | 2.41 | 3.24 |
|  | *Tetramorium noratum* | 2.51 | 2.89 |
|  | *Tetramorium* sp1 | 2.96 | 3.17 |
|  | *Tetramorium* sp2 | 2.85 | 2.95 |
|  | *Tetramorium* sp3 | 3.12 | 3.42 |
|  | *Tetramorium* sp4 |  | 3.29 |
|  | *Tetramorium* sp7 | 2.53 | 2.86 |
|  | *Vollenhovia* sp1 | 3.08 | 2.71 |
|  | *Vollenhovia* sp2 | 3.46 | 3.95 |
|  | *Vollenhovia* sp5 |  | 2.62 |
| **Ponerinae** | *Anochetus graeffei* | 3.42 | 3.64 |
|  | *Anochetus incultus* |  | 3.30 |
|  | *Anochetus* sp2 | 3.19 | 3.30 |
|  | *Cryptopone* sp1 | 3.93 |  |
|  | *Hypoponera* sp1 | 3.11 | 3.37 |
| **Subfamily** | **Species** | **Unlogged Forest** | **Logged Forest** |
| **Ponerinae (contd.)** | *Hypoponera* sp2 | 3.50 | 3.04 |
|  | *Hypoponera* sp3 | 3.35 | 3.60 |
|  | *Hypoponera* sp5 | 3.34 | 3.74 |
|  | *Hypoponera* sp6 | 3.86 | 3.94 |
|  | *Hypoponera* sp7 | 3.27 | 3.34 |
|  | *Hypoponera* sp8 | 3.20 | 3.60 |
|  | *Leptogenys near mutabilis* | 2.89 | 3.37 |
|  | *Leptogenys* sp4 | 3.78 |  |
|  | *Leptogenys* sp5 | 3.19 |  |
|  | *Leptogenys* sp6 | 3.56 |  |
|  | *Leptogenys* sp7 | 2.95 |  |
|  | *Leptogenys* sp8 | 3.17 |  |
|  | *Leptogenys* sp9 | 3.42 | 3.13 |
|  | *Myopias* sp1 |  | 3.25 |
|  | *Odontomachus rixosus* | 2.69 | 3.08 |
|  | *Odontoponera transversa* | 3.00 | 3.32 |
|  | *Odontoponera* sp2 | 2.83 | 2.93 |
|  | *Pachycondyla leeuwenhoeki* | 2.85 | 3.08 |
|  | *Pachycondyla rubra* | 3.25 | 3.22 |
|  | *Pachycondyla tridentata* | 2.90 | 2.87 |
|  | *Pachycondyla* sp2 | 3.18 |  |
|  | *Pachycondyla* sp3 | 3.22 | 3.33 |
|  | *Pachycondyla* sp7 | 3.52 |  |
|  | *Pachycondyla* sp9 | 3.56 |  |
|  | *Ponera* sp2 |  | 3.70 |
|  | *Ponera* sp3 | 3.25 | 3.67 |
|  | *Ponera* sp4 | 3.31 | 3.63 |
|  | *Ponera* sp6 | 3.33 | 3.38 |
| **Proceratiinae** | *Discothyrea* sp1 | 2.93 |  |
|  | *Discothyrea* sp2 | 3.45 | 3.33 |
|  | *Proceratium* sp1 | 2.60 | 2.60 |
|  | *Proceratium* sp2 | 3.80 |  |
| **Pseudomyrmecinae** | *Tetraponera* sp1 | 2.00 |  |
